# Spectral and Kinetic Properties of Radicals Derived from Oxidation of Quinoxalin-2-One and Its Methyl Derivative

**DOI:** 10.3390/molecules191119152

**Published:** 2014-11-19

**Authors:** Konrad Skotnicki, Julio R. De la Fuente, Alvaro Cañete, Krzysztof Bobrowski

**Affiliations:** 1 Centre of Radiation Research and Technology, Institute of Nuclear Chemistry and Technology, Dorodna 16, Warszawa 03-195, Poland; E-Mail: k.skotnicki@ichtj.waw.pl; 2 Departamento de Quimica Orgánica y Fisicoquímica, Facultad de Ciencias Químicas y Farmacéuticas, Universidad de Chile, Casilla 223, Santiago 1, Chile; E-Mail: jrfuente@ciq.uchile.cl; 3 Departamento de Química Orgánica, Facultad de Química, Pontificia Universidad Católica de Chile, Casilla 306, Correo 22, Santiago, Chile; E-Mail: acanetem@uc.cl

**Keywords:** quinoxalin-2-ones, radicals, pulse radiolysis, hydroxyl radicals, azide radicals

## Abstract

The kinetics and spectral characteristics of the transients formed in the reactions of ^•^OH and ^•^N_3_ with quinoxalin-2(1*H*)-one (Q), its methyl derivative, 3-methylquinoxalin-2(1*H*)-one (3-MeQ) and pyrazin-2-one (Pyr) were studied by pulse radiolysis in aqueous solutions at pH 7. The transient absorption spectra recorded in the reactions of ^•^OH with Q and 3-MeQ consisted of an absorption band with λ_max_ = 470 nm assigned to the OH-adducts on the benzene ring, and a second band with λ_max_ = 390 nm (for Q) and 370 nm (for 3-MeQ) assigned, *inter alia*, to the N-centered radicals on a pyrazin-2-one ring. The rate constants of the reactions of ^•^OH with Q and 3-MeQ were found to be in the interval (5.9–9.7) × 10^9^ M^–1^·s^–1^ and were assigned to their addition to benzene and pyrazin-2-one rings and H-abstraction from the pyrazin-2-one nitrogen. In turn, the transient absorption spectrum observed in the reaction of ^•^N_3_ exhibits an absorption band with λ_max_ = 350 nm. This absorption was assigned to the N-centered radical on the Pyr ring formed after deprotonation of the respective radical cation resulting from one-electron oxidation of 3-MeQ. The rate constant of the reaction of ^•^N_3_ with 3 MeQ was found to be (6.0 ± 0.5) × 10^9^ M^–1^·s^–1^. Oxidation of 3-MeQ by ^•^N_3_ and Pyr by ^•^OH and ^•^N_3_ confirms earlier spectral assignments. With the rate constant of the ^•^OH radical with Pyr (*k* = 9.2 ± 0.2) × 10^9^ M^–1^·s^‒1^, a primary distribution of the ^•^OH attack was estimated nearly equal between benzene and pyrazin-2-one rings.

## 1. Introduction

Quinoxaline-2-one derivatives have recently received much attention owing both to their biological properties and pharmaceutical applications [1,2]. These derivatives are particularly interesting since some of them showed a variety of pharmacological properties, such as antimicrobial [3,4,5,6], antiviral [7], antifungal [5,8,9], anxiolytic [10], analgesic [9], antiinflammatory [6], antithrombotic [11,12], and antitumor [13,14,15,16] activities. For instance, as far as the antitumor activity is concerned, recent studies have proved that quinoxalin-2-ones restrain activity of the Platelet-Derived Growth Factor Receptors (PDGFR) [17], proteins responsible for growth and division of cells. A cell cycle regulation, due to the inhibition of cyclin-dependent kinases (Cdk), is another example of anticancer activity of quinoxalinones [18]. Their receptors belong to the tyrosine kinase family and are common targets in cancer treatment. Structure Activity Relationship (SAR) studies have revealed that quinoxalin-2-ones derivatives bound to protein receptors (e.g., in Cdk) are generally located close to the adenosine triphosphate (ATP) binding pocket [13,17]. The fact that these compounds are bound in very specific position in proteins may have serious consequences in their interactions with either amino acid residues or radicals derived from them. Certain amino acids residues—tyrosine (Tyr), tryptophan (Trp), and cysteine (Cys)—are particularly vulnerable to oxidation. Therefore, the radical cations derived from quinoxalin-2-ones can modify these amino acids that are reasonably good electron donors and can be oxidized to tyrosyl (TyrO^•^), tryptophyl (TrpN^•^), and thiyl (CysS^•^) radicals, respectively. On the other hand, these radicals are reasonably good electron acceptors and can potentially act as oxidants of quinoxalin-2-ones intercalated in a protein matrix.

Quinoxalin-2-one-based compounds also exhibit an inhibition activity on certain enzymes involved in HIV-1 [1,19,20], and on matrix metaloproteinases (MPP) [21]. A growth in MPP activity and a simultaneous depletion of natural metalloproteinase inhibitors are observed in multiple sclerosis (SM).

**Figure 1:**
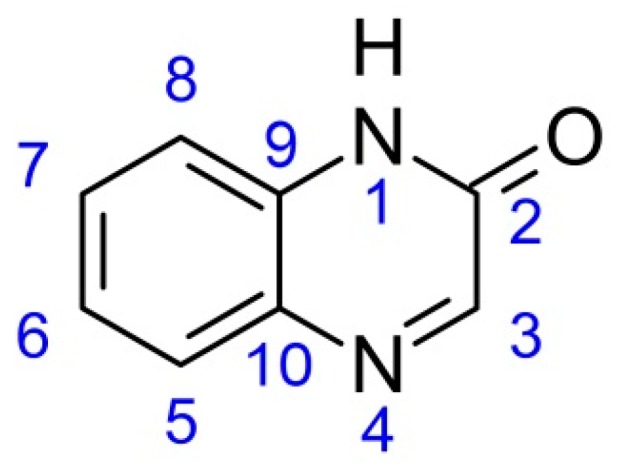
General structure of quinoxalin-2-ones.

A key factor that is decisive in their biological activity is substitution at the carbon-3 in the pyrazine ring and at the carbons 6 and 7 in the benzene ring of the primary quinoxalin-2-one structure (Figure 1). Nearly all biologically active derivatives are substituted in those specific positions.

Based on the computer modeling and “*in vitro*” studies, quinoxalin-2-ones have been proposed as potential drugs in treatments of various diseases [1]. Therefore, they are used as the scaffolds of many drugs for the treatment of several diseases. Some of them (based on 1-methyl-7-nitro-4-(5-(piperidin-1-yl)pentyl)-3,4-dihydroquinoxalin-2(1*H*)-one) are in medical trials or in animal laboratory tests [22]. On the market, there are currently two quinoxalin-2-one-based drugs available, called Spasmium and Spadon, that are permitted for use in humans [23]. They are based on caroverine hydrochloride (1-[2-(diethylamino)ethyl]-3-[(4-methoxyphenyl)methyl]-2(1*H*)-quinoxal- inone monohydrochloride), and are used as antispasmodic agents and in a treatment of inner ear diseases. Since Reactive Oxygen Species (ROS) are supposed to be involved in the pathogenesis of the inner ear, caroverine can act as a ROS scavenger. It was found that the reaction of caroverine hydrochloride with ^•^OH radicals occurs with a high rate constant [24].

Surprisingly, there are only a few reports about radical processes involving quinoxalin-2-one derivatives. Some derivatives were found to initiate free radical polymerization by electron transfer from *N*-phenylglycine [25,26]. Their photoreduction by amines leads to the corresponding stable products dihydroquinoxalin-2-ones or the reductive dimers depending on the substituent in position 3 of the pyrazine ring [27]. Formation of tricyclic azetidines resulting from [2 + 2] cycloadditions of quinoxalin-2-ones with alkenes was also reported [28,29,30,31].

The photophysical and photochemical behavior of 1-methyl-3-phenylquinoxalin-2-one and 3-phenylquinoxalin-2-one in the presence of amines has been reported in some selected organic solvents (acetonitrile, methanol and hexane) [32,33]. Spectral and kinetic characteristics of the intermediate species: triplet ion-radical pairs (Q^•−^/amine^•+^) and hydrogenated neutral radicals (QH^•^) have been obtained by laser flash photolysis [32]. Calculated spectra using quantum mechanical semi-empirical AM1, PM3, and ZINDO/S approach were found to be in agreement with the experimental spectra [32]. Recently, the stable products resulting from the photoreduction of six 7-substituted- 3-methyl-quinoxalin-2(1*H*)-one derivatives (substituents: H_3_CO-, H_3_C-, F-, H-, CF_3_-, CN-) by α-amino-type radicals derived from *N*-phenylglycine (PhNHCH_2_^•^) in acetonitrile solutions have been identified. Moreover, spectral and kinetic characteristics of the intermediate species: triplets (^3^Q), radical anion (Q^•−^) and hydrogenated neutral radicals (QH^•^) have been obtained by laser flash photolysis in the presence of DABCO and *N*-phenylglycine (NPG) [34].

The electrochemical studies of three 3-methyl-quinoxalin-2(1*H*)-ones: 3-methyl- quinoxalin-2(1*H*)-one, 3,6,7-trimethylquinoxalin-2(1*H*)-one and 7-amino-3-methylquinoxalin-2(1*H*)-one showed that the pyrazine ring is the electroactive center undergoing two-electron reduction [35].

The examples given above strongly indicate a necessity to gain a comprehensive and systematic knowledge about spectral and kinetic properties of radicals and radical ions derived from quinoxalin-2-ones. In particular, there is no information about the radicals and radical cations forming during oxidation of these compounds. Therefore, in the current work we have investigated oxidation processes in quinoxalin-2(1*H*)-one (Q) and its methyl derivative, 3-methylquinoxalin-2(1*H*)-one (3-MeQ) in aqueous solutions at pH 7, starting from the primary transients formed by radiation-induced hydroxyl (^•^OH) and azide (^•^N_3_) radicals. These studies comprise spectral and kinetic properties of transients formed, including an assessment of the ^•^OH and ^•^N_3_ reactivity by determination of the respective second-order rate constants with quinoxalin-2-ones.

## 2. Results and Discussion

**Scheme 1:**
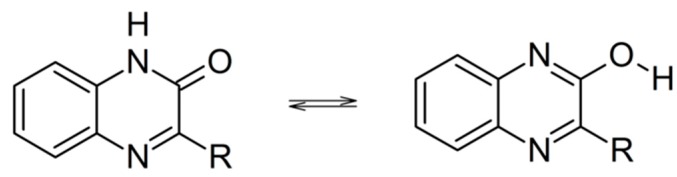
Tautomerization of quinoxalin-2-ones.

Quinoxalin-2-ones can potentially coexist with the tautomeric isomers called 2-hydroxyquinoxalines (Scheme 1). However, crystal and molecular structure studies [36], matrix-isolation studies [37], as well as theoretical calculations have shown that the keto isomers are the dominant forms [37,38].

**Scheme 2:**
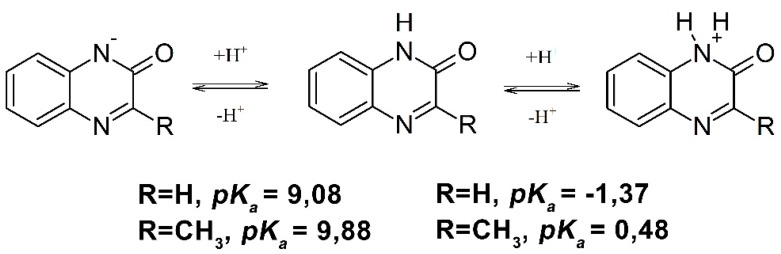
Acid-base equilibria of quinoxalin-2-ones.

Moreover, depending on the pH, quinoxalin-2-ones in aqueous solutions exist in three forms that are involved in acid-base equilibria with the reported respective *pK*_a1_ and *pK*_a2_ values (Scheme 2) [39,40].

### 2.1. Reactions of ^•^OH

#### 2.1.1. Quinoxalin-2(1*H*)-One (Q)

A transient absorption spectrum, obtained 4 μs after the electron pulse in N_2_O-saturated solutions containing 0.1 mM of Q at pH 7, exhibited a distinct absorption band with λ_max_ = 390 nm and a second, very broad and flat absorption band in the 450–500 nm range with no marked maximum. The respective *G* × ε are *G* × ε_390_ = 1.0 × 10^–3^ dm^3^·J^–1^·cm^–1^ and *G* × ε_450-500_ = 0.5 × 10^–3^ dm^3^·J^–1^·cm^–1^. Moreover, a bleaching signal was observed in the 340–370 nm range with λ_max_ = 360 nm, which corresponds to the absorption maximum of Q in the ground state. There was also an intensive and broad nondescript absorption band with no distinct λ_max_ < 280 nm and *G* × ε_280_ = 2.25 × 10^–3^ dm^3^·J^–1^·cm^–1^ (Figure 1, curve a).

The rate of formation, followed at wavelengths 390, and 470 nm, fits to a single exponential. The measured pseudo-first-order rate constants show a linear dependence on the concentration of Q (Figure 1, right inset) with the slopes representing the respective second-order rate constants for the formation of transient(s) resulting from the reaction of ^•^OH radicals with Q. The calculated second-order rate constants *k*_390_ = (7.3 ± 0.7) × 10^9^ M^–1^·s^–1^ and *k*_470_ = (9.3 ± 0.3) × 10^9^ M^–1^·s^–1^ are similar within the experimental error limit of ±15% in pulse radiolysis, and are close to those measured for diffusion-controlled reactions.

**Figure 2:**
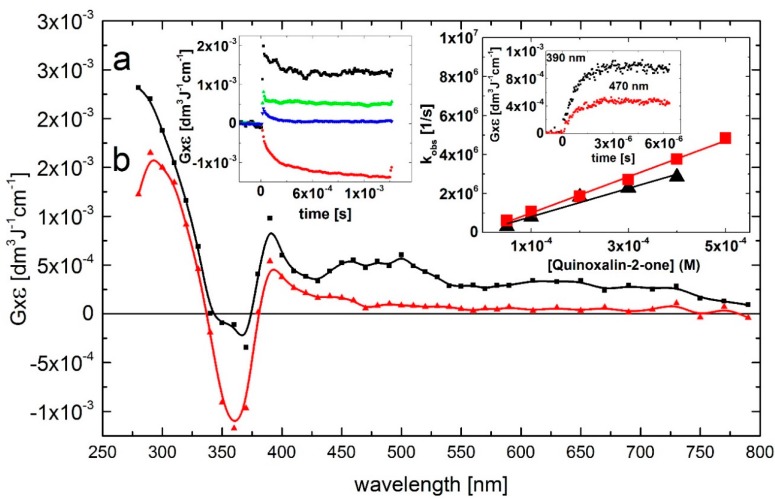
Transient absorption spectra (uncorrected for the ground-state absorption) recorded in N_2_O-saturated aqueous solution containing 0.1 mM Q at pH 7. Spectrum **a**, time delay 4 μs; spectrum **b**, time delay 500 μs. Insets: (left) Time profiles representing decays at λ = 280 (■), 390 (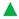), and 470 nm (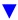) and bleaching at λ = 360 nm (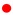); (right) Plots of the observed pseudo-first-order rate constants of the formation of the 390-nm (▲) and 470 nm (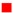) absorption (*k*_obs)_ as a function of Q at pH 7. Inset of right inset: Time profiles representing growths at λ = 390 and 470 nm at 0.1 mM concentration of Q.

The broad absorption band in the 450–500 nm range decayed in a second-order reaction with the first τ_1/2_ = 90 μs measured at λ_max_ = 470 nm. The decay kinetic trace showed a nearly complete decay of the transient, that reached a residual value at 1.5 ms at a very low level of *G* × ε_470_ = 1 × 10^–4^ dm^3^·J^–1^·cm^–1^. An interesting feature concerns the decay kinetic traces recorded at wavelengths 280 and 390 nm. They are characterized by distinctly different time profiles (Figure 2, left inset) than that at λ_max_ = 470 nm. The decay kinetic traces at λ_max_ = 290 and 390 nm fell to plateaus within 500 μs and 60 μs, respectively, which persisted within the 1.5 ms time domain at relatively high levels of *G* × ε_290_ = 1.3 × 10^–3^·dm^3^·J^–1^·cm^–1^ and *G* × ε_390_ = 0.5 × 10^–3^ dm^3^·J^–1^·cm^–1^. This complex behavior suggests that secondary products (some of them can be stable, vide infra) are formed characterized by a transient spectrum with a strong UV band having λ_max_ = 290 nm and *G* × ε_290_ = 2.0 × 10^–3^ dm^3^·J^–1^·cm^–1^ and a weaker absorption band with λ_max_ = 390 nm and *G* × ε_390_ = 0.5 × 10^–3^ dm^3^·J^–1^·cm^–1^ (Figure 2, curve b).

At this point in the exposition, we will not attempt to assign the species responsible for the 380/390-nm and 470 absorption bands. However, considering various possible sites of the ^•^OH radical attack, one can easily note that different transients are responsible for the presence of these two bands. This issue will be discussed later.

#### 2.1.2. 3-Methylquinoxalin-2(1*H*)-One (3-MeQ)

**Figure 3:**
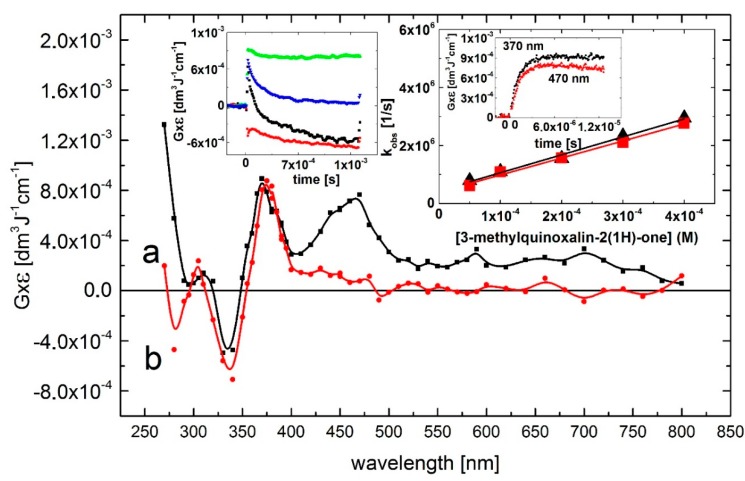
Transient absorption spectra (uncorrected for the ground-state absorption) recorded in N_2_O-saturated aqueous solution containing 0.1 mM 3-MeQ at pH 7. Spectrum **a**, time delay 4 μs; spectrum **b**, time delay 1 ms. Insets: (left) Time profiles representing decays at λ = 280 (■), 370 (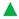), and 470 nm (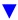) and bleaching at λ = 330 nm (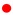); (right) Plots of the observed pseudo-first-order rate constants of the formation of the 370-nm (▲) and 470 nm (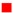) absorption (*k*_obs)_ as a function of 3-MeQ concentration at pH 7. Inset of right inset: Time profiles representing growths at λ = 390 and 470 nm at 0.1 mM concentration of 3-MeQ. Bottom right inset: Absorption spectrum recorded in N_2_O-saturated aqueous solution containing 0.2 mM 3-MeQ at pH 7 after γ-irradiation with a dose of 30 Gy.

The reaction of ^•^OH radicals with 3-MeQ was investigated in order to check to what extent substitution at the carbon-3 by a methyl group in the pyrazine ring (see Scheme 2) influences the ^•^OH reactivity and the spectral and kinetic characteristics of the transients formed. The transient absorption spectrum, obtained 4 μs after the electron pulse in N_2_O-saturated solutions containing 0.1 mM of 3-MeQ at pH 7 exhibited two absorption bands with λ_max_ = 370 and 470 nm with *G* × ε_370_ = 9.5 × 10^–4^ dm^3^·J^–1^·cm^–1^ and *G* × ε_470_ = 8.5 × 10^–4^ dm^3^·J^–1^·cm^–1^. Contrary to Q, a much more pronounced maximum at λ = 470 nm was observed. A bleaching signal was also observed, however, in the 320–350 nm range with λ_max_ = 330 nm, which corresponds to the absorption maximum of 3-MeQ in the ground state. There was also an intensive and broad nondescript absorption band with no distinct λ_max_ < 280 nm with *G* × ε_280_ = 1.4 × 10^–3^ dm^3^·J^–1^·cm^–1^ (Figure 3, curve a).

The calculated second-order rate constants for the formation of transient(s), resulting from the reaction of ^•^OH radicals with 3-MeQ measured at wavelengths 370 and 470 nm (Figure 3, right inset) *k*_370_ = (6.2 ± 0.3) × 10^9^ M^–1^·s^–1^ and *k*_470_ = (5.9 ± 0.3) × 10^9^ M^–1^·s^–1^ are slightly lower in comparison to those measured at pH 7.0 for Q (*vide supra*).

Similar to Q, there was an absorption band with λ_max_ = 470 nm that decayed in a second-order reaction with a first τ_1/2_ = 85 μs. The decay kinetic trace showed also a nearly complete decay of the transient, fall to a residual absorption at 1.5 ms at a very low level of *G* × ε_470_ = 0.5 × 10^–4^ dm^3^·J^–1^·cm^–1^ (Figure 3, left inset). However, the decay kinetic trace recorded at λ = 290 nm had a featured different from that of Q. At this wavelength there was a strong bleaching signal observed in 3-MeQ, instead of the plateau observed earlier for Q. On the other hand, the decay kinetic trace at λ_max_ = 370 nm reached a plateau at *G* × ε_370_ = 8.5 × 10^–4^ dm^3^·J^–1^·cm^–1^within 250 μs, and persisted within the 1.5 ms time domain. This complex behavior suggests that secondary products (some of them can be stable, *vide* Figure 3, bottom right inset) were formed characterized by a transient spectrum with a weak band having λ_max_ = 320 nm and *G* × ε_320_ = 2.0 × 10^–4^ dm^3^·J^–1^·cm^–1^ and a stronger absorption band with λ_max_ = 370 nm and *G* × ε_370_ = 8.5 × 10^–4^ dm^3^·J^–1^·cm^–1^ (Figure 3, curve b).

At this point in the exposition, we will not attempt to assign the species responsible for the 380/390-nm and 470 absorption bands in quinoxalin-2(1*H*)-ones. Concerning the decay kinetics of the transients, clear differences depending on the wavelength were registered. As shown in Figure 2 and Figure 3 the absorption bands with λ_max_ = 470 nm decayed nearly to zero on the millisecond time domain whereas the absorption bands with λ_max_ = 370–390 nm and 280 nm were stable. This fact indicates, considering various possible sites of the ^•^OH radical attack on the quinoxalin-2(1*H*)-ones, the formation of different transients. This issue will be discussed below.

#### 2.1.3. Assignment of the Species Responsible for the 370/390-nm and 470 Absorption Bands

**Scheme 3:**
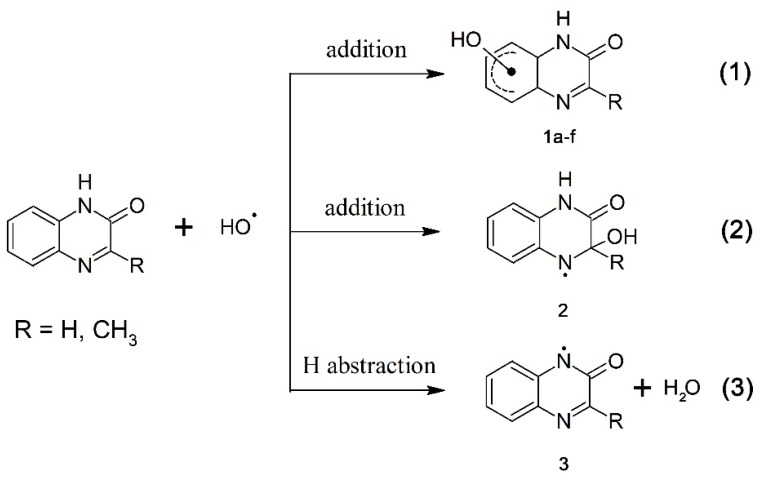
Reaction pathways of ^•^OH radicals with quinoxalin-2-ones.

**Scheme 4:**
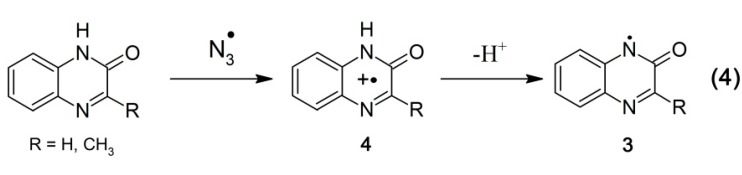
Oxidation of quinoxalin-2-ones by ^•^N_3_ radicals.

It is well established that ^•^OH radicals are highly reactive toward heterocyclic and aromatic compounds. This was also confirmed in this work since the rate constants for the reactions of ^•^OH radicals with quinoxalin-2(1*H*)-ones were in the range (5.9–9.7) × 10^9^ M^–1^·s^–1^ *i.e.*, with nearly diffusion controlled rates. Aromatic compounds usually react with ^•^OH radicals simultaneously via several competing pathways, e.g., abstraction of an H atom, addition to an aromatic ring and a double bond to form adducts at different positions. Therefore, besides formation of ^•^OH adducts on the benzene ring (**1a**–**f**) (reaction 1, Scheme 3) and on the pyrazin-2-one ring (**2**) (reaction 2, Scheme 3), also an H-abstraction from the pyrazin-2-one nitrogen leading to the N-centered radical (**3**) (reaction 3, Scheme 3) is conceivable. The latter radical can be also formed via deprotonation of the N-centered radical cation (**4**) formed during one-electron oxidation of quinoxalin-2-ones (Scheme 4, reaction 4).

##### 2.1.3.1. Oxidation by ^•^N_3_ Radicals

To verify this hypothesis, the reaction of Q and 3-MeQ with ^•^N_3_ radicals was investigated. The ^•^N_3_ radical is an oxidant that reacts exclusively by one-electron transfer. Since ^•^N_3_ radical is not reactive with benzene [41], one can expect that oxidation will exclusively occur on the pyrazin-2-one ring (Scheme 4).

**Figure 4:**
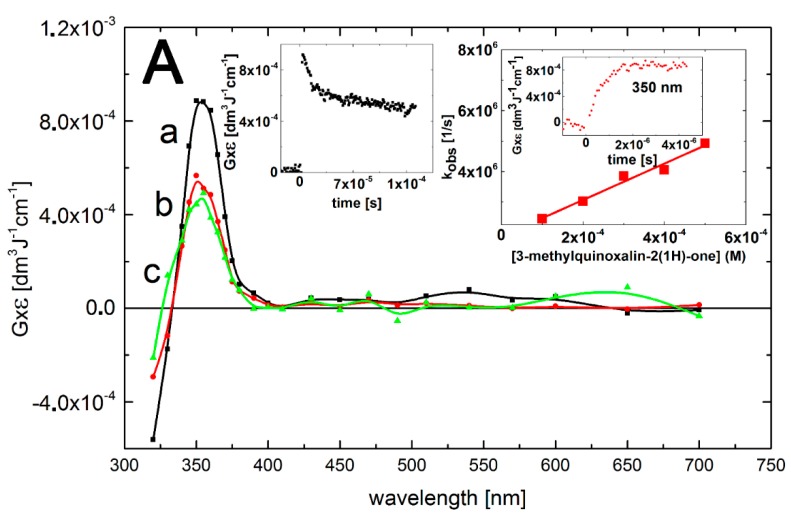

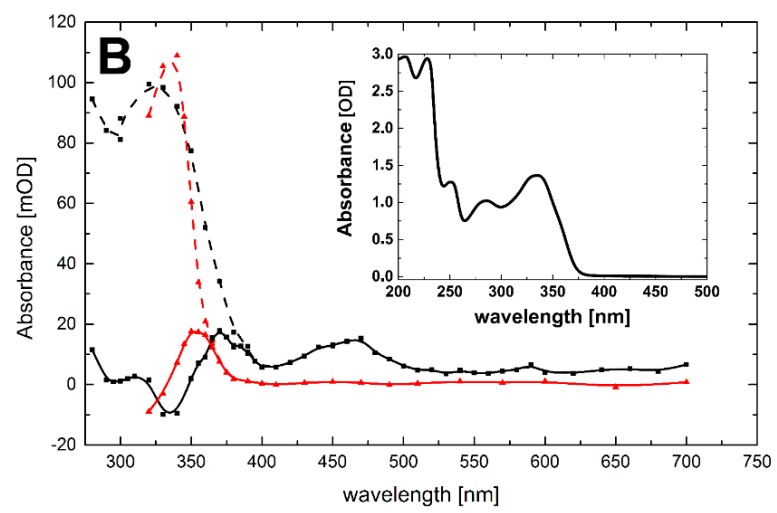
(**A**) Transient absorption spectra (uncorrected for the ground-state absorption) recorded in N_2_O-saturated aqueous solution containing 0.1 mM 3-MeQ and 0.1 M NaN_3_ at pH 7. Spectrum **a**, time delay 4 μs (■); spectrum **b**, time delay 50 μs (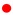); spectrum **c**, time delay 1.5 ms (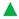). Insets: (left) Time profile representing decay at λ = 350 nm; (right) Plot of the observed pseudo-first-order rate constants of the formation of the 350-nm absorption (*k*_obs)_ as a function of 3-MeQ concentration at pH 7. Inset of right inset: Time profile representing growth at λ = 350 nm at 0.1 mM concentration of 3-MeQ; (**B**) Transient absorption spectra uncorrected (solid lines), and corrected for the ground-state absorption (dashed lines) recorded 4 μs after the electron pulse in N_2_O-saturated aqueous solution at pH 7 and containing 0.1 mM of 3-MeQ, without (■), and with 0.1 M NaN_3_ (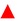). Inset: Ground-state absorption spectrum of 3-MeQ in aqueous solutions at pH 7.

A transient absorption spectrum recorded at 4 μs after the electron pulse in N_2_O-saturated aqueous solutions containing 0.1 mM of 3-MeQ and 0.1 M of N_3_^–^ at pH 7 exhibited a narrow and distinct absorption band with λ_max_ = 350 nm and *G* × ε_350_ = 9.0 × 10^–4^ dm^3^·J^–1^·cm^–1^ (Figure 4, curve a).

Interestingly, the absorption band with λ_max_ = 470 nm was not observed which may suggest that the OH-adducts to the benzene ring **1a**–**f** (Scheme 3, reaction 1) are responsible for this absorption band. The maximum absorbance at λ_max_ = 350 nm was reached at about 4 μs (Figure 4A, right inset), after which, the absorption band decreases without the change of the shape (Figure 4A, curves b and c). However, the decay of the 350-nm absorption band showed biphasic kinetics (Figure 4A, left inset). The first part of kinetic trace displayed a decay with a half-life of t_1/2_ = 25 μs, followed by a very slow decay that nearly reached a plateau within 1.5 ms. The spectral and kinetic features observed in the analogous solutions containing Q were very similar.

The rate constant for the reaction of ^•^N_3_ radicals with 3-MeQ was determined from a linear dependence of the pseudo-first order build-up of the absorbance at 350 nm on the 3-MeQ concentration. The slope of this plot gives the bimolecular rate constant *k*_350_(^•^N_3_ + 3-MeQ) = (6.0 ± 0.5) × 10^9^ M^–1^·s^–1^ (Figure 4A, right inset). The high reactivity of ^•^N_3_ radicals with 3-MeQ arises from the lower oxidation potential of 3-MeQ as compared with the reduction potential of ^•^N_3_ radicals (E° = +1.33 V *vs.* NHE) [42]. This is not surprising in view of the very low ionization potential of 3-MeQ calculated in water [43]. The short-lived absorption band with λ_max_ = 350 nm (Figure 4A, curve a) observed during the reaction of 3-MeQ with ^•^N_3_ radicals (Scheme 4, reaction 4) is tentatively assigned to the deprotonated form of the N-centered radical cation **3**. However, the location of the λ_max_ is blue-shifted by 20 nm in comparison to the λ_max_ observed for the reaction of ^•^OH radicals (Figure 3, curve a). A major obstacle for performing a reliable comparison of the transient absorption spectra is due to depletion of 3-MeQ ground state, which absorbs at this spectral region as depicted in the inset of Figure 4B. In order to overcome this problem, both experimental spectra were corrected for bleaching in the spectral range 280–380 nm. Both corrected spectra are characterized by strong absorption maxima bands with λ_max_ = 320 and 340 nm for transients resulting from ^•^OH and ^•^N_3_ reactions, respectively (Figure 4B). One may thus suggest that the N-centered radicals **3** are responsible for the short-lived and narrow absorption band with λ_max_ = 340 nm. On the other hand the broad 320-nm absorption band can be tentatively assign to the superposition of absorption bands of other isomer(s) of ^•^OH adducts on the benzene ring **1** formed in reaction 1 (Scheme 3), ^•^OH adducts on the pyrazin-2-one ring **2** formed in reaction 2 (Scheme 3), and of the *N*-centered radicals **3** formed in reaction 3 (Scheme 3). The last reaction is generally considered as a slower one in comparison to those comprising ^•^OH addition to the aromatic rings. Therefore, this reaction might be of minor importance, however, cannot be eliminated.

##### 2.1.3.2. Oxidation of Pyrazin-2-One (Pyr) by ^•^OH and ^•^N_3_ Radicals

**Scheme 5:**
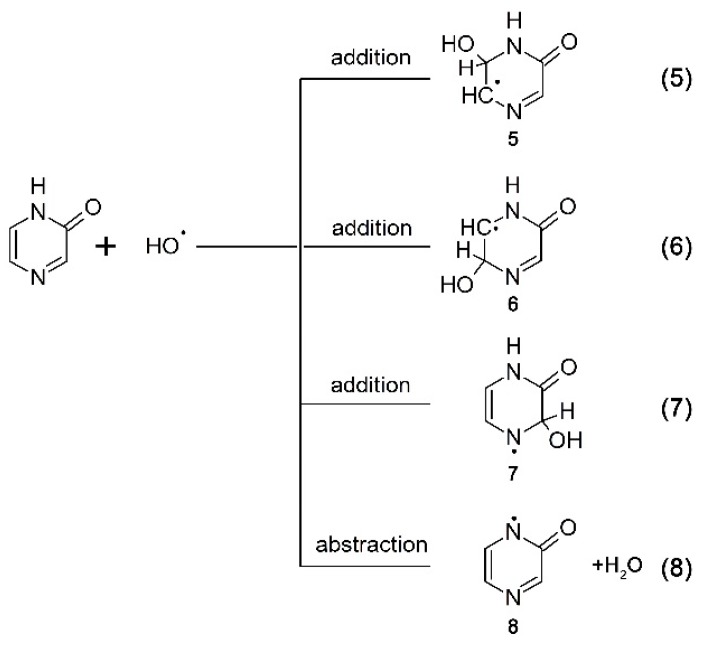
Reaction pathways of ^•^OH radicals with pyrazin-2-one.

To verify the last assumption, the reactions of ^•^OH and ^•^N_3_ radicals with Pyr were investigated. One might expect that ^•^OH radicals will react with double bonds (Scheme 5, reactions 5–7) to form adducts at different positions **5**–**7** and abstract H-atom from N1 to form the N-centered radicals **8** (Scheme 5, reaction 8).

**Figure 5:**
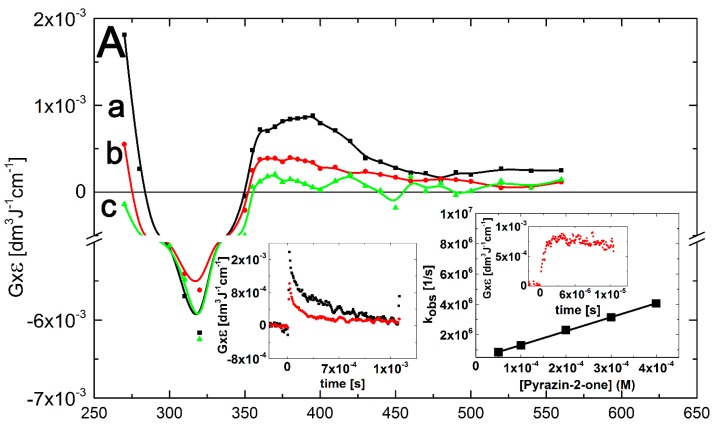

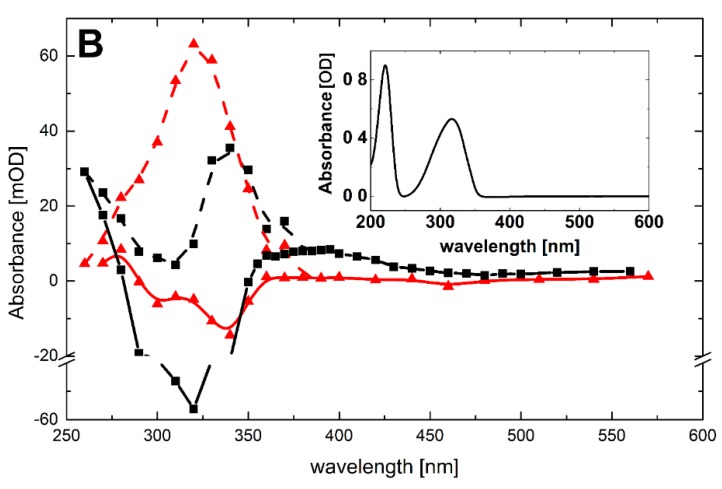
(**A**) Transient absorption spectra (uncorrected for the ground-state absorption) recorded in N_2_O-saturated aqueous solution containing 0.1 mM Pyr at pH 7. Spectrum **a**, time delay 4 μs (■); spectrum **b**, time delay 50 μs (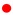); spectrum **c**, time delay 1.5 ms (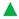). Insets: (left) Time profiles representing decays at λ = 270 nm (●) and 390 nm (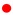); (right) Plot of the observed pseudo-first-order rate constants of the formation of the 390-nm absorption (*k*_obs)_ as a function of Pyr concentration at pH 7. Inset of right inset: Time profile representing growth at λ = 390 nm at 0.1 mM concentration of Pyr; (**B**) Transient absorption spectra uncorrected (solid lines), and corrected for the ground-state absorption (dashed lines) recorded 4 μs after the electron pulse in N_2_O-saturated aqueous solution at pH 7 and containing 0.1 mM of Pyr, without (■), and with 0.1 M NaN_3_ (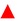). Inset: Ground-state absorption spectrum of Pyr in aqueous solutions at pH 7.

A transient absorption spectrum, obtained 4 μs after the electron pulse in N_2_O-saturated solutions containing 0.1 mM of Pyr at pH 7, exhibited a very broad and rather flat absorption band with λ_max_ = 380–390 nm with *G* × ε_390_ = 9.0 × 10^–4^ dm^3^·J^–1^·cm^–1^ after which the absorption decreased without change in its shape (Figure 5A, curves a, b and c).

It is noteworthy that an absorption band with λ_max_ = 470 nm did not form, contrary to N_2_O-saturated solutions containing Q (see Figure 2, curve a) and 3-MeQ (see Figure 3, curve a) when ^•^OH was used as an oxidant. However, this observation is similar to that when ^•^N_3_ was used as an one-electron oxidant, instead (see Figure 4A, curve a). This observation is compatible with the earlier conclusion that the existence of OH-adduct(s) in a benzene ring are responsible for the 470-nm absorption band. A very strong bleaching signal is also observed in the 280–350 nm range with λ_max_ = 320 nm, which corresponds to the absorption maximum of Pyr in the ground state. There was also an intensive and broad nondescript absorption band with no distinct λ_max_ < 260 nm and *G* × ε_260_ = 1.8 × 10^–3^ dm^3^·J^–1^·cm^–1^ (Figure 5A, curve a).

The rate of formation, followed at λ_max_ = 390 nm, fits to a single exponential, and the measured pseudo-first order rate constants showed a linear dependence on the concentration of Pyr (Figure 5A, right inset) with a slope representing the respective second-order rate constants for the formation of transient(s) resulting from the reaction of ^•^OH with Pyr. The calculated second-order rate constant (*k*_390_(^•^OH + Pyr) = (9.2 ± 0.2) × 10^9^ M^–1^·s^–1^ is close within experimental error to those measured for Q and 3-MeQ (*vide* supra). Again, a major obstacle for performing a reliable comparison is due to depletion of the Pyr ground state that strongly absorbs at this spectral region (Figure 5B, inset). From the results presented so far, it can be concluded that the transient spectra represents superpositions of several absorption bands due to the presence of various transients **5**–**8** resulting from the reaction pathways depicted in Scheme 5.

**Scheme 6:**
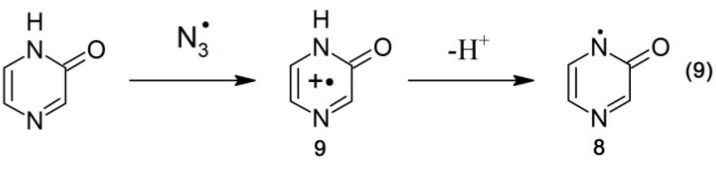
Oxidation of pyrazin-2-one by ^•^N_3_ radicals.

On the other hand, ^•^N_3_ radicals are expected to react exclusively by one electron transfer (Scheme 6, reaction 9).

A transient absorption spectrum recorded at 4 μs after the electron pulse in N_2_O-saturated solutions containing 0.1 mM of Pyr and 0.1 M of N_3_^–^ at pH 7 did not exhibit any absorption, except for a very weak absorption at λ_max_ = 280 nm (Figure 5B, red solid line). However, the presence of a bleaching signal observed in the 290–350 nm range indicates that ^•^N_3_ radicals were reactive with Pyr. Surprisingly, a bleaching signal was substantially weaker than that observed when ^•^OH was used as the oxidant (Figure 5B, black solid line). Since the concentrations of ^•^OH and ^•^N_3_ reacting with Pyr were similar in the experimental conditions used, this might indicate that the lower apparent depletion of the Pyr ground state was due to the formation of a transient absorbing in the same spectral range. Moreover, this transient was formed with a higher yield compared to the system when ^•^OH was used as an oxidant. This is not surprising based on Scheme 5 and Scheme 6. When Pyr was oxidized by ^•^N_3_ radicals, the formation of *N*-centered radicals **8** was the only possible reaction pathway (Scheme 6, reaction 9). On the other hand, when ^•^OH radicals reacted with Pyr, the formation of N-centered radicals **8** represents one of the four possible reaction pathways (Scheme 5, reaction 8).

As previously, in order to overcome the obstacle due to depletion of Pyr ground state, the experimental spectra were corrected for bleaching in the spectral range 260–360 nm. Both corrected spectra are characterized by strong absorption maxima located at λ_max_ = 340 and 320 nm for transients resulting from ^•^OH and ^•^N_3_ reactions, respectively. One may thus suggest that N-centered radicals **8** are responsible for the absorption band with λ_max_ = 320 nm.

##### 2.1.3.3. Primary Distribution of the ^•^OH Attack on the Quinoxalin-2(1*H*)-One Molecule

There are two competitive reaction channels for the reaction of ^•^OH radicals with quinoxalin-2(1*H*)-one molecules: attack on the benzene and pyrazin-2-one rings, respectively. Taking the respective rate constants of the reactions of ^•^OH radicals with benzene (k = (7.8 ± 1.1) × 10^9^ M^−1^·s^–1^) [44], and with pyrazin-2-one (k = (9.2 ± 0.2) × 10^9^ M^–1^·s^–1^) (this work, vide supra) one can easily calculate nearly equal primary distribution of the ^•^OH attack on the main residue targets, i.e., benzene and pyrazin-2-one rings. This is just a rough approximation neglecting possible conjugation of the nitrogen atoms with the benzene ring. However, with this approximation taken together with an assumption that most of the ^•^OH radicals attacking the benzene ring form Q5OH and 3-MeQ5OH (vide infra), their initial concentration formed in the system after absorbing a dose of 20 Gy (vide Experimental Section) can be estimated as 5.6 μM. Based on the equation 2k = 1/τ_1/2_ [A_0_], and taking the respective τ_1/2_ values (vide Subsection 2.1.1 and Subsection 2.1.2), one can calculate the rate constants of the second-order decay reactions for Q5OH and 3MeQ5OH as 2.0 × 10^9^ M^–1^·s^–1^ and 2.1 × 10^9^ M^–1^·s^–1^, respectively. These values are in a good accordance with the rate constants of the second-order decay reactions of hydroxycyclohexadienyl radicals.

##### 2.1.3.4. OH-Adducts on a Benzene Ring

In order to get the information for the most likely sites of the ^•^OH attack on a benzene ring, Mulliken charge densities (q_M_) on carbon atoms, and enthalpies of the OH-adducts formation (Δ*H*_f_) on a benzene ring were calculated. The lowest deficiencies of a negative charge were found on C5, C7, and C9 atoms, both for Q and 3-MeQ (Table 1).

**Table 1:** Mulliken charge densities in Q and 3-MeQ molecules.

Compound	N1	C2	O	C3	N4	C5	C6	C7	C8	C9	C10
**Q**	0.106	0.235	0.365	0.099	0.905	−0.099	−0.137	−0.052	−0.144	−0.076	−0.144
**3-MeQ**	0.105	0.225	0.361	0.132	0.019	−0.018	−0.138	−0.047	−0.147	−0.069	−0.126

The most favorable enthalpies of the OH-adducts formation (Δ*H*_f_) were found for the addition at C5, C7, and C8 atoms for both compounds (Table 2).

**Table 2:** Enthalpies of the OH-adducts formation (Δ*H*_f_) in a benzene ring of Q and 3-MeQ.

Compound	C5	C6	C7	C8
**Q**	−28.49	−26.19	−**29.30**	−29.92
**3-MeQ**	−**37.78**	−35.08	−37.09	−38.63

(Δ*H*_f_) are given in kcal·mol^–1^.

The data listed in Table 1 and Table 2, taken together, lead to the conclusion that positions C7 and C5 in a benzene ring are most likely the preferable sites for the ^•^OH addition in Q and 3-MeQ, respectively.

With these information, a better insight can be obtained from spectroscopically relevant active electronic transitions and oscillator strengths for the respective OH-adducts at position 5 and 7 in Q and 3-MeQ and for N-centered radicals in 3-MeQ. They are summarized in Table 3.

**Table 3:** Spectral parameters of the OH-adducts in Q and 3-MeQ and of the N-centered radical in 3-MeQ.

Q5OH		Q7OH		3-MeQ5OH		3-MeQ7OH		3-MeQ1N	
λ nm	*f*	λ nm	*F*	λ nm	*f*	λ nm	*f*	λ nm	*f*
523.5	0.029	673.6	0.020	537.6	0.032	683.0	0.038	476.7	0.023
**421.5**	**0.137**	635.0	0.037	**420.5**	**0.109**	638.6	0.022	465.1	0.016
374.5	0.081	413.5	0.016	**374.9**	**0.103**	418.9	0.016	**382.7**	**0.133**
**337.7**	**0.134**	**357.3**	**0.081**	**345.0**	**0.157**	**354.9**	**0.081**	326.0	0.062
302.4	0.030	322.0	0.034	304.3	0.040	**323.2**	**0.082**	324.5	0.017
279.0	0.025	**317.4**	**0.071**	286.5	0.024	310.5	0.010	309.0	0.127
265.0	0.017	279.3	0.040	266.2	0.020	280.6	0.025	281.1	0.011
231.1	0.029	277.9	0.013	241.2	0.012	268.8	0.039	248.0	0.156
227.2	0.017	260.7	0.044	238.2	0.012	252.6	0.267	233.9	0.708
225.6	0.017	247.3	0.253	226.7	0.015	247.7	0.166	223.4	0.135

The compatibility between the experimentally observed and the calculated absorption spectra for the respective OH-adducts at positions 5 and 7 in Q and 3-MeQ (Table 3) is reasonably good. The calculated electronic transitions for the OH-adducts at position 5 reproduce fairly well the main feature of the experimental spectra recorded for Q and 3-MeQ, *i.e.*, the presence of the absorption bands > 400 nm. In addition, the calculated electronic transitions <400 nm for the OH adducts at position 5 and 7 clearly show that these adducts contribute strongly to the absorption band with λ_max_ = 320 nm observed for 3-MeQ (Figure 4B, corrected black curve), together with N-centered radicals **3** depicted in Scheme 3. The latter conclusion is further supported by a comparison with the absorption band recorded when ^•^N_3_ was used as an oxidant (Figure 4B, corrected red curve)). In this case, only the N-centered radicals (**3**) depicted in Scheme 6 can contribute to the experimental absorption spectrum in this region. The most intense calculated electronic transition for N-centered radicals **3** differs by 30 nm (Figure 4B, corrected red curve). However, it reproduces fairly well the main feature of the experimental absorption spectrum recorded for 3-MeQ, *i.e.*, presence of a narrow absorption band with a strong and distinct maximum (Figure 4A,B).

## 3. Experimental Section

### 3.1. Materials

Quinoxalin-2(1*H*)-one (2-quinoxalinol, Q) and pyrazin-2-one (2-hydroxypyrazine, Pyr) were purchased from Aldrich (Poznan, Poland) and used without further purification. Nitrous oxide (N_2_O) >98% was from Messer (Warsaw, Poland). 3-Methylquinoxalin-2(1*H*)-one (3-methyl-2-quinoxalinol, 3-MeQ) was prepared by the classical reaction of the corresponding *o*-phenyldiamine (1 mmol) by adding dropwise methyl pyruvate (1.2 mmol) and triethylamine (3 mmol) in ethanol. A detailed description of the synthesis, purification and spectral characterization has been given elsewhere [34]. All solutions were made with water triply distilled provided by a Millipore Direct-Q 3-UV system. The pH was adjusted by the addition of NaOH. Prior to irradiation, the samples were purged gently with N_2_O for 30 min. per 50 mL volume before experiments. The typical concentration of solutions in pulse radiolysis experiments was 0.1 mM of quinoxalin-2-ones and pyrazin-2-one at pH 7, unless otherwise specified.

### 3.2. Uv-Vis Spectrophotometry

The absorption spectra were recorded with a V-670 UV-vis spectrophotometer (JASCO, Cremella, Italy) using a 1 cm optical path length cell. Water without additives was used as a reference sample. An aliquot of 1 mL was taken to measure the absorption spectrum.

### 3.3. Pulse Radiolysis

Pulse radiolysis experiments were performed with the INCT LAE 10 MeV linear accelerator (city, country) with a typical pulse length of 8 ns. A detailed description of the experimental setup has been given elsewhere along with the basic details of the equipment and the data collection system [45]. Absorbed doses per pulse were on the order of 20 Gy (1 Gy = 1 J·kg^–1^). Dosimetry was based on N_2_O-saturated solutions containing 10^–2^ M KSCN, taking a radiation chemical yield of G = 0.635 μmol J^–1^ and a molar absorption coefficient of 7580 M^–1^·cm^–1^ at 472 nm for the (SCN)_2_^•–^ radical [46]. Experiments were performed with a continuous flow of sample solutions at room temperature (~23 °C).

### 3.4. γ-Radiolysis

γ-Irradiations were carried out using the ^60^Co γ-rays source GC 5000 (India) in the Institute of Nuclear Chemistry and Technology (Warsaw, Poland). The dose rate was equal to 98.4 Gy·min^–1^, as determined by the Fricke dosimetry. All irradiations were performed at room temperature. Samples were purged gently with N_2_O for 20 min before irradiation.

### 3.5. Spectral Analysis of Time-Resolved Spectra

In any time window, following the electron pulse, the absorbance of the solution is related to the radiation chemical yield (*G*) and the molar absorption coefficients (ε) by the formula (i):

(i) $$G\times \varepsilon \left(\lambda j\right)=\;\Delta A\left(\lambda j\right)\frac{\epsilon_{472}\;G{\left(SCN\right)}_{2}^{•-}}{{\text{ΔΑ}}_{472}}$$

which are how spectra are displayed in Figure 2, Figure 3, Figure 4A and Figure 5A

Equation (i) is convenient way to normalize the absorbance ΔA(λ_j_) to the absorbed dose, the inverse of which is proportional to ε_472_*G*((SCN)_2_^•–^)/Δ*A*_472_ from thiocyanate dosimetry, where ε_472_ is the molar absorption coefficient of (SCN)_2_^•–^ at 472 nm, *G*((SCN)_2_^•–^) is the radiation chemical yield of the (SCN)_2_^•–^ radicals, and Δ*A*_472_ represents the observed absorbance change at λ = 472 nm in the thiocyanate dosimeter. The *Gε*(λ_j_) are in turn related to the underlying transients via Beer’s Law since Δ*A* = log(I_0_/I) is the absorbance of the sample.

### 3.6. Water Radiolysis

During water radiolysis, e^–^_aq_, ^•^OH, and ^•^H were generated with radiation chemical yields of 0.28, 0.28, and 0.06 μmol·J^–1^, respectively [47]. In order to convert e^–^_aq_ into ^•^OH radicals all solutions were purged with N_2_O. The e^–^_aq_ reacted with N_2_O according to reaction 10 with *k*_10_ = 9.1 × 10^9^ M^–1^·s^–1^ [48]. Reaction (1) nearly doubled the amount of ^•^OH radicals available for reactions with substrates:

e^–^_aq_ + N_2_O + H_2_O → O^•–^ + N_2_ (+ H_2_O) → ^•^OH + N_2_ + OH^–^ (1)

Since the radiation chemical yield of ^•^H atoms at pH 7 was roughly 10-fold lower than that of ^•^OH radicals, the contribution of transient absorption spectra of products from ^•^H reactions with substrates was neglected.

The reactions of ^•^N_3_ radicals with substrates were investigated in N_2_O-saturated solutions containing 0.1 M NaN_3_. Under these experimental conditions, ^•^OH radicals were quantitatively scavenged by azide anions (N_3_^–^) according to reaction (2) with *k*_11_ = 1.2 × 10^10^ M^–1^·s^–1^ [41].

^•^OH + N_3_^–^ → ^•^N_3_ + OH^–^ (2)

### 3.7. Calculations

Mulliken charge densities (q_M_) on atoms in Q and 3-MeQ, and enthalpies of the OH-adducts formation (∆*H*_f_) were calculated on the PM3 semi-empirical level, using Hypercube HyperChem 8.0.3 software. The geometry of the adducts was first optimized using Molecular Mechanics MM+ method followed by geometrical optimization at the PM3 level using a charge = 0 and multiplicity = 2, and the UHF approximation. Calculated spectra, employing formerly optimized the PM3 geometries for the OH-adducts, were obtained by using ZINDO/S RHF Single Pont CI calculations, and considering the first ten unoccupied and ten occupied MO. A larger number of absorption lines in the high-energy region beyond the range of measurements were obtained, however, without affecting the spectral region of interest in this study.

## 4. Conclusions

In the present studies unambiguous evidence is provided for an ^•^OH radical addition both to benzene and pyrazin-2-one rings in quinoxalin-2(1*H*)-one (Q) and its derivative 3-methylquinoxalin-2(1*H*)-one (3-MeQ). A primary distribution of the ^•^OH attack was found nearly equal between benzene and pyrazin-2-one rings. The second-order rate constants for the reaction of ^•^OH radicals with Q and MeQ were measured, both being nearly equal to diffusion controlled rates. On the other hand, the ^•^N_3_ radical, as an one-electron oxidant, reacts with Q and 3-MeQ forming an N-centered radical on the pyrazin-2-one ring. The same intermediate was also formed following the direct hydrogen abstraction from the >N-H group by ^•^OH radicals. The second-order rate constant for the reaction of ^•^N_3_ radicals was found to be similar to those measured involving ^•^OH radicals with 3-MeQ, suggesting its rather low oxidation potential.
